# SMILE-like lesion in the anal canal

**DOI:** 10.4322/acr.2021.289

**Published:** 2021-05-25

**Authors:** Aravind Sekar, Surinder Singh Rana, Anand Bardia

**Affiliations:** 1 Post Graduate Institute of Medical Education and Research Department of Histopathology, Chandigarh, India; 2 Post Graduate Institute of Medical Education and Research Department of Gastroenterology, Chandigarh, India

**Keywords:** Anal canal, Adenocarcinoma, Polyp, Carcinoma in Situ

## Abstract

Stratified mucin-producing intraepithelial lesion (SMILE) is an intraepithelial lesion with overlapping features of the high-grade squamous intraepithelial lesion (HSIL) and adenocarcinoma in situ (AIS). Currently, it is well described in the cervix. We present a case showing similar SMILE-like lesions in the polypectomy specimen from the anal canal along with invasive adenocarcinoma components. This lesion showed an immuno-profile characteristic of a SMILE lesion described in the cervix, such as p63 negativity, high ki67 index, and nuclear positivity for p16. It might be arising from the Human papillomavirus prone transitional region of the anal canal as described in the cervix. However, we could not assure this association and etiological link due to insufficient material in the formalin-fixed paraffin-embedded block. Notwithstanding, we strongly suggest that the HPV is the main driver for this SMILE-like lesion similar to what is described in the cervix. To our knowledge, this is the first case report of a SMILE lesion in the anal canal. Further studies will be required to elucidate the underlying pathogenetic mechanism of SMILE-like lesions described in the anal canal.

## INTRODUCTION

A stratified mucin-producing intraepithelial lesion (SMILE) is an intraepithelial lesion described first in the cervix by Park et al.^1^ It has a unique morphology characterized by the overlapping features of the high-grade squamous intraepithelial lesion (HSIL) and adenocarcinoma in situ (AIS). It differs from classical HSIL alone by (i) the presence of diffuse intracytoplasmic mucin and (ii) lack of p63, p40 immuno-expressions, and (iii) AIS by the presence of stratification and absence of glands within the lesion.^2^ And, it can also progress to an invasive carcinoma with morphological features identical to those in SMILE.^3^ The Pathogenesis of SMILE lesion is associated with High-risk Human Papillomavirus infection (HPV 16,18 and others).^4^ It arises from the distinct HPV-prone squamocolumnar junction cells in the transformation zone of the cervix. As SMILE lesion shares similar HPV induced pathogenetic mechanisms with other intraepithelial lesions, it can coexist with HSIL, AIS, squamous cell carcinoma, and adenocarcinoma.^5^

This unique intraepithelial lesion is well described in the cervix and also observed in the vulva^6^ and recently in the penis.^7^ And it is not described elsewhere in the body. We observed a distinct intraepithelial lesion in the transitional region of the anal canal, showing true stratification and diffuse intracytoplasmic mucin vacuoles. It shows similar morphological and immunohistochemical characteristics of the SMILE lesion described in the cervix. SMILE-like lesion observed in the anal canal is new-fangled for us and, as far as we know, not described in the literature till now.

## CASE REPORT

A 40-year-old man presented to the gastroenterology outpatient department complaining of pain after defecation for 3 months. There was no history of bleeding or mucoid discharge per rectum. Rectal examination showed unremarkable perianal skin, but growth at the fingertip was palpable. Subsequent endoscopic examination of the large intestine showed friable polypoidal growth in the anal canal. The remaining parts of the colon up to the terminal ileum were normal. Abdominal contrast-enhanced computed tomography (CECT) showed a well-defined polyp measuring 18x13x12 mm in the anterior wall of the anorectal junction. There was no definite muscular layer and serosa involvement noted on the pelvic MRI, neither locoregional lymphadenopathy was identified. Perirectal and perianal fat were normal. Serum CEA level was 2.7 ng/ml (Reference Range; RR; 0-4.0 ng/ml). Anti-HIV was negative. Polypectomy was done, and tissue was submitted for histopathological examination.

**Figure 1:**
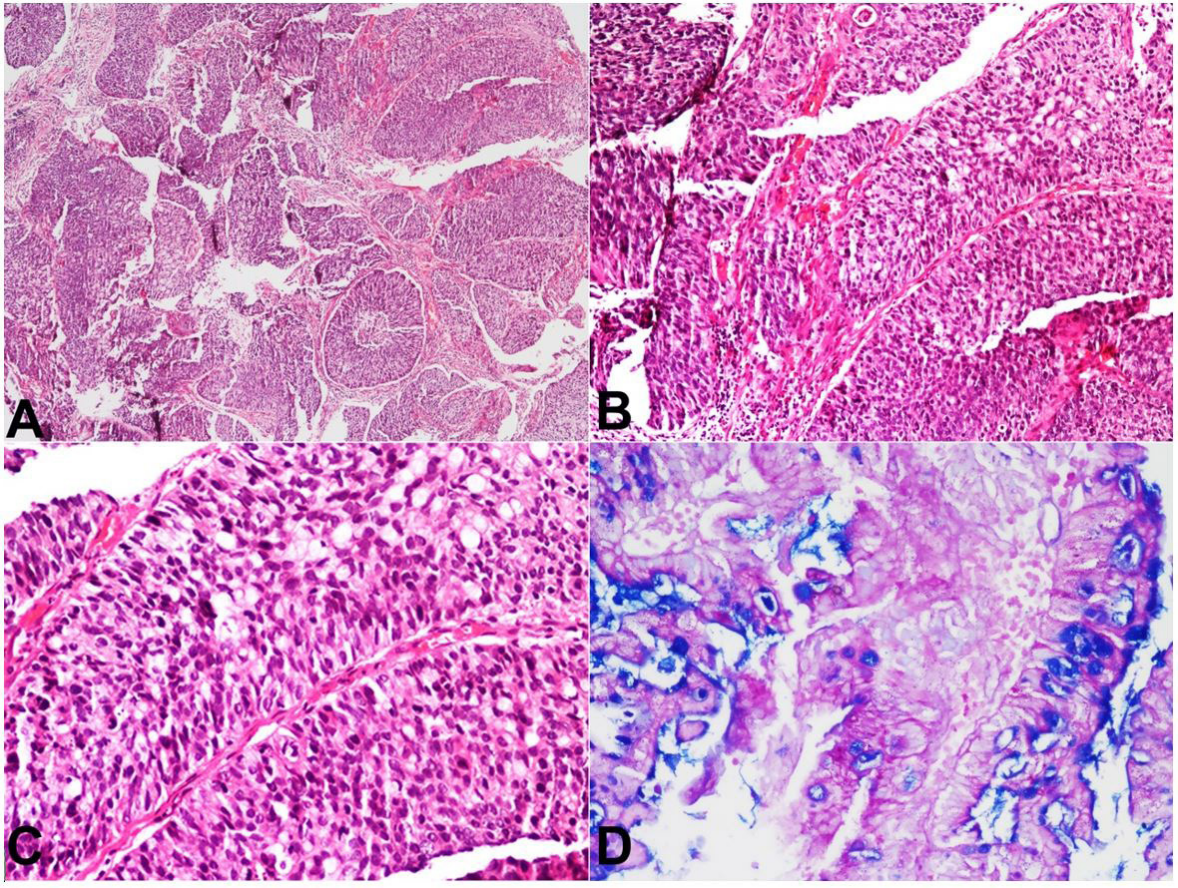
Photomicrographs of the biopsy. **A** – Fragmented polypoidal tissue with an invasive tumor arranged in papillary configuration, nests and trabeculae. (H&E, x 40); **B** and **C** – Papillae with SMILE like lesion [SIL] displaying full thickness dysplastic stratified epithelial cells with diffuse abundant intracytoplasmic mucin displacing nucleus to periphery. (H&E, x 100 and x200, respectively); **D** – Intracytoplasmic mucin is highlighted on PAS-AB stain. (x 200).

On microscopy, it showed fragmented polypoidal tissue with papillary folds lined by variably thickened truly stratified epithelial cell layers with a maximum of 20 cell thickness (Figure 1A, B). The stratified layers of epithelial cells exhibited full thickness dysplasia with marked nuclear atypia, hyperchromasia, and increase number of mitotic figures, resembling a high-grade squamous intraepithelial lesion (Figure 1C). However, the epithelial cells in the stratified layers had also shown intracytoplasmic mucin vacuoles. Due to the abundance of intracytoplasmic mucin in a few cells, the nucleus was displaced to the periphery and gave a signet ring-like morphology. PAS-AB highlighted intracytoplasmic mucin in the stratified epithelial cell layers (Figure 1 D). There was no gland formation within these stratified layers. Also, adjacent fragments showed invasive components with tumor cells arranged in solid nests with central comedonecrosis, tubules, and trabecular patterns (Figure 22B).

**Figure 2:**
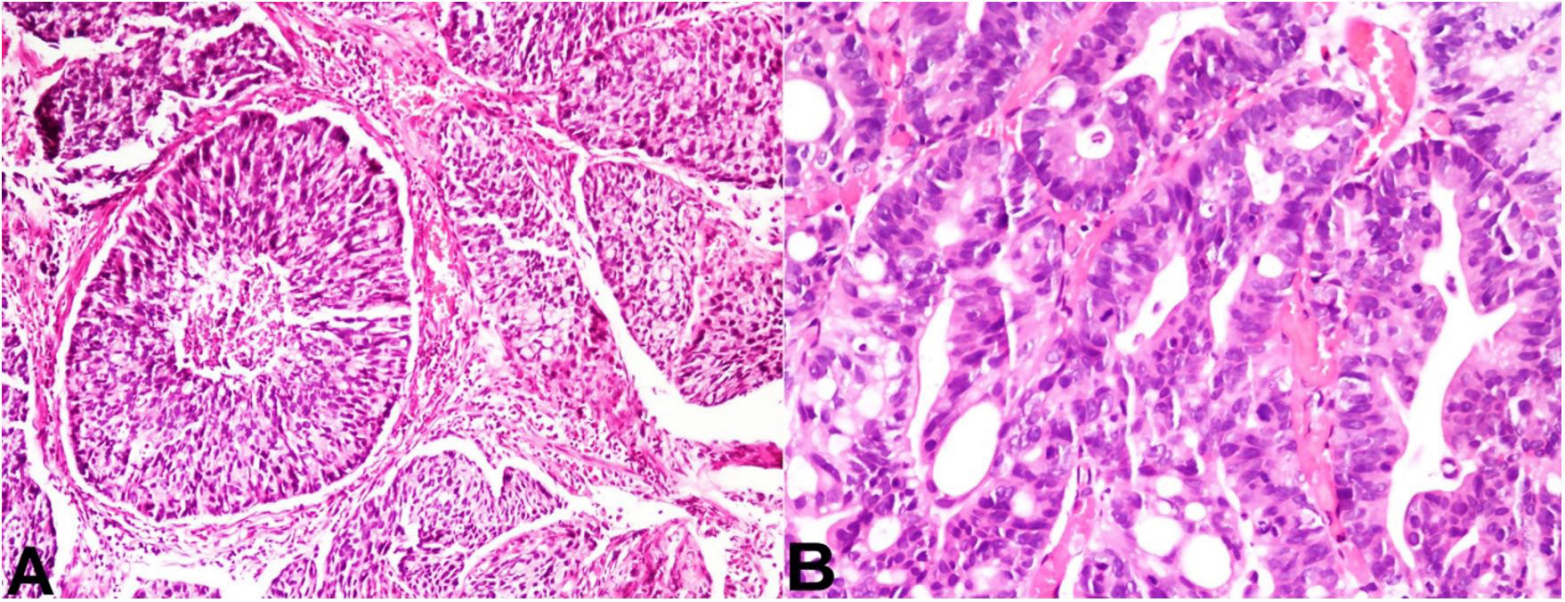
Photomicrographs of the biopsy. **A** and **B** – Usual type adenocarcinoma with complex glandular architecture] and nests of tumor cells with central comedo like necrosis are also seen in invasive components. (H&E, x 200, both).

**Figure 3:**
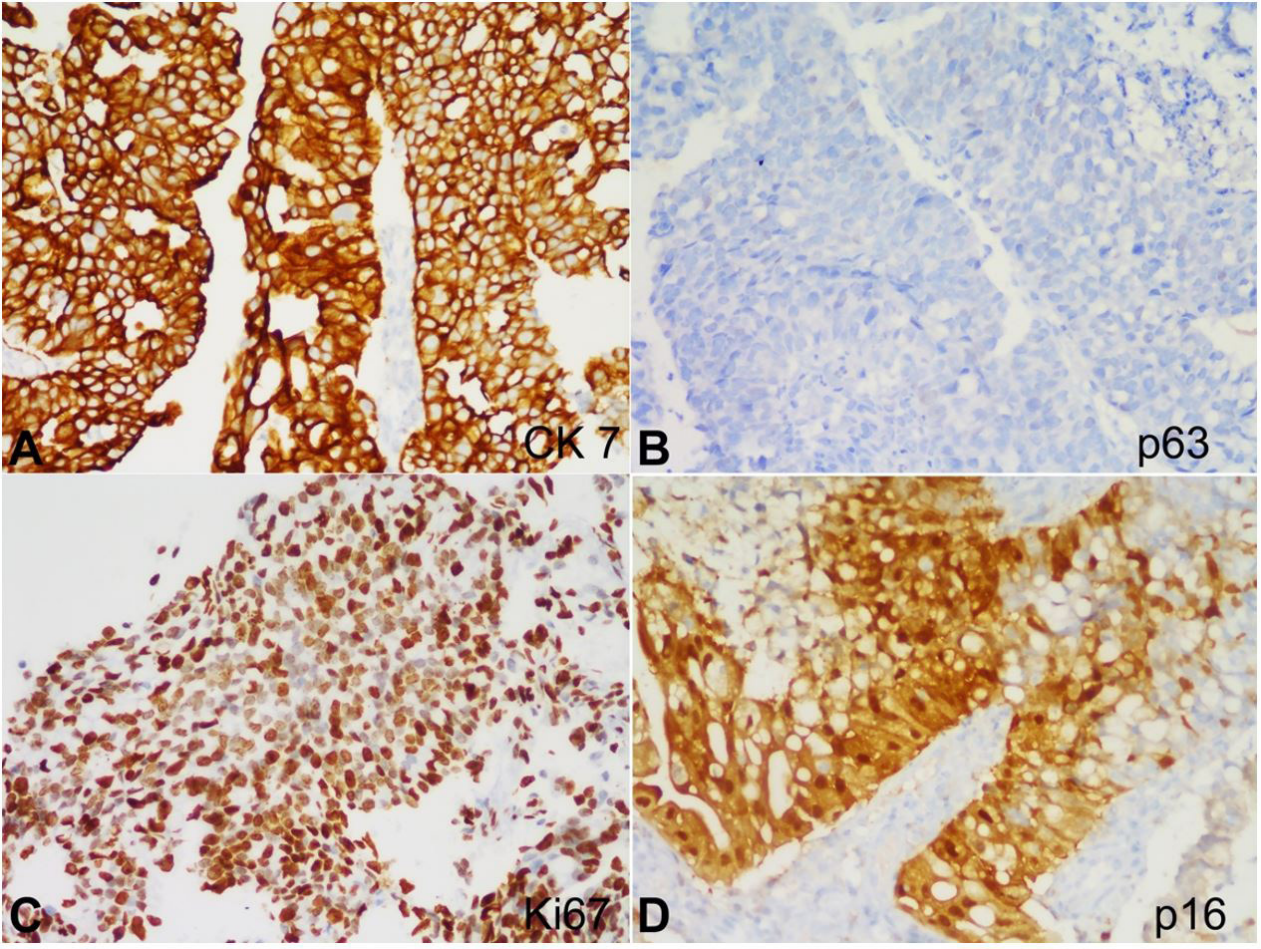
Photomicrographs of the biopsy. **A** – strong membranous positivity for CK 7; **B** – negative expression for p63; **C** – high ki67 index; **D** – nuclear expression for p16. (all pictures x 200).

On the immunohistochemistry, these stratified cells showed strong immunoreactivity for CK7 and negative expression for p63, excluding the possibility of a high-grade squamous intraepithelial lesion. Furthermore, these cells showed strong nuclear expression for p16, and Ki 67 index was 80% (Figure 3AD).

Though the usual type adenocarcinoma component was noted in a few fragments in the invasive areas, the observation of truly stratified layers with intracytoplasmic mucin is unusual in the anal canal and not documented in the literature. Unfortunately, due to insufficient material in the formalin-fixed paraffin-embedded block, we could not perform the HPV-ISH or genetic sequencing for the human papillomavirus.

After the polypectomy, whole-body FDG PET CT was performed, which showed mild diffuse FDG uptake in the anorectal region. The patient further received 16 cycles of chemotherapy for the residual tumor. Furthermore, now he is on the 8-month follow-up without symptoms.

## DISCUSSION

The in-situ carcinoma lesion with a true stratification, full-thickness dysplasia with intracytoplasmic vacuoles observed in this case is a distinct intraepithelial lesion not described in the anal canal till now. This lesion is very similar to SMILE lesions of the cervix. Histologically, the anal canal can be divided into three parts such as the external zone lined by squamous epithelium, the transitional zone, and the inner colorectal zone.^8^ The transitional zone is more prone to human papillomavirus infection, as is the cervix, and can give rise to squamous cell carcinoma and adenocarcinoma. Nearly half of the anal adenocarcinoma arising from the transitional zone was etiologically linked to a high-risk of HPV infection.^9^

During the initial evaluation of this case with the H&E stain, the considered differential diagnosis was (i) a high-grade squamous intraepithelial lesion, (ii) a conventional adenoma giving rise to adenocarcinoma, and (iii) a rare transitional epithelial type of fistula-associated anal canal adenocarcinoma. However, the presence of diffuse intracytoplasmic mucin and lack of intercellular bridges, and no expression for p63 on immunostaining, excluded the possibility of the high-grade squamous intraepithelial lesion. Though the usual type adenocarcinoma component is noted in one fragment showing invasion, the presence of true stratification, even up to a maximum of 20 cell thickness in the in-situ lesion, makes this lesion different from a conventional adenoma. The nuclear stratification giving the pseudostratified appearance and the focal intracellular mucin can be seen in the transitional epithelial type of fistula- associated anal canal adenocarcinoma. ^10^ ^,^ ^11^ However, the observation of true stratification up to 20 layers and the presence of diffuse intracytoplasmic mucin vacuoles in the index case, besides the absence of fistulous tract on rectal examination, make this lesion distinct.

Despite the lack of the HPV-ISH availability, the immuno-profile observed in the in-situ component, such as strong nuclear expression for p16, lack of expression for p63, and high ki67 index, in the index case, is very similar to the immuno-profile of SMILE lesion described in the cervix. Probably, similar to the SMILE lesion of the cervix, the lesion observed in our case might arise from the reserve cells or urothelial-like cells in the transitional region. On searching for SMILE-like lesions outside the cervix in the literature, we found that McCluggage et al.,^6^ reported 2 cases of vulval intraepithelial neoplasia with mucinous differentiation, and Michael et al.,^7^ observed an analogue of stratified mucin producing intraepithelial lesion in the penis. So, maybe SMILE-like lesion is not restricted to the cervix; it can be seen in other areas that are more susceptible to HPV infection.

## CONCLUSION

Lack of material for molecular analysis is the major pitfall of this study. However, the observation of a SMILE-like lesion in the anal canal is a new finding for us, and this lesion exhibited an immuno-profile as in the cervix. It might be arising from reserve cells or urothelial-like cells of HPV-prone transitional zone. We believe the lesion observed in our case is very similar to the SMILE lesion described in the cervix. To our knowledge, this is the first case that describes SMILE-like lesion in the anal canal. Unquestionably, genetic studies will require further to determine the underlying molecular characterization and pathogenesis of SMILE-like lesion (SIL) in the anal canal.
